# Improved knowledge and reported practice regarding sexually transmitted infections among healthcare providers in rural Vietnam: a cluster randomised controlled educational intervention

**DOI:** 10.1186/s12879-014-0646-5

**Published:** 2014-12-04

**Authors:** Pham Thi Lan, Ho Dang Phuc, Nguyen Quynh Hoa, Nguyen Thi Kim Chuc, Cecilia Stålsby Lundborg

**Affiliations:** Hanoi Medical University, Hanoi, Vietnam; Global Health (IHCAR), Department of Public Health Sciences, Karolinska Institutet, Stockholm, Sweden; Institute of Mathematics, VAST, Hanoi, Vietnam; National Cancer Hospital, Hanoi, Vietnam

**Keywords:** STI, Healthcare provider, Intervention, Knowledge, Reported practice, Vietnam

## Abstract

**Background:**

Healthcare providers (HCPs) play a critical role in controlling the spread of sexually transmitted infections (STI) through early and accurate diagnosis, appropriate treatment and prevention counselling. This study aimed to assess the effectiveness of an educational intervention about STI on knowledge and reported practice among HCPs and to explore which determinants may influence the intervention’s effects.

**Methods:**

A cluster randomized controlled educational intervention was carried out in a rural district, Vietnam. 32 communes of the district were randomized into two arms, with 160 HCPs in an STI intervention arm and 144 in a control arm. The STI intervention comprised interactive training with basic STI knowledge, case scenarios, and poster distribution. Questionnaires to evaluate knowledge and reported practice were completed three times: before, during and after the intervention. Correct answer was scored as 1; “do not know”, incorrect answer was scored as 0. Univariate and multilevel multivariate analyses were applied.

**Results:**

Of the maximum 56 points, the mean knowledge score increased significantly in the STI intervention arm and in the control arm post-intervention (37.2 to 48.4, and 32.7 to 41.7, respectively). In multivariate regression analysis, knowledge improvement in the intervention arm was significantly higher than that in the control arm (regression coefficient = 2.97, p = 0.008). Other factors which positively influenced the increase in knowledge were being between 35 and 50 years old, having intermediate professional training, being a pharmacist or working at a village level (regression coefficient: 2.81, 4.43, 5.53 and 7.91, respectively). Post-intervention, the mean reported practice score increased significantly in the STI intervention arm (from 17.6 to 21.8) and insignificantly in the control arm (maximum 36 points). Factors which positively influenced the increase in reported practice were being between 35 and 50 years old, having intermediate professional training, or working at a pharmacy/drugstore (regression coefficient: 2.15, 3.33 and 3.22, respectively).

**Conclusions:**

This study indicates that an educational intervention including interactive training and multi-faceted interventions may be effective in improving STI knowledge and reported practice of HCPs at grassroots level, particularly among pharmacists, HCPs who work in villages or pharmacies/drugstores, and who initially have low STI knowledge.

**Electronic supplementary material:**

The online version of this article (doi:10.1186/s12879-014-0646-5) contains supplementary material, which is available to authorized users.

## Background

Sexually transmitted infections (STI) have a negative impact on the health, economy and quality-of-life of individuals, as well as whole communities. The World Health Organization estimates that there are 499 million new cases of the four most common curable STI (trichomonasis*,* chlamydial infection, gonorrhea and syphilis) each year worldwide [1]. Furthermore, millions of viral STI cases also occur annually, and are attributable mainly to the human immunodeficiency virus (HIV), herpes, human papilloma virus and hepatitis B [2]. Improved case management of STI has been scientifically proven to reduce the incidence of HIV infection in the general population [3],[4]. In low-income countries, STI management is usually inadequate [5]-[7] and STI control programmes often fail for various reasons [8]. It has been shown that healthcare providers (HCPs) play an important role in reducing the burden of STI through effective prevention and management [9]. In addition, studies have shown positive impact of continuing medical education or educational interventions on improvement of HCPs’ knowledge and/or practices [10],[11], STI management [12],[13] and patient outcomes [14].

In Vietnam, curable STI are common among high-risk groups [15],[16] and not uncommon in the general population [17]. Misconceptions and low knowledge on STI among people in the community [18],[19] and HCPs [20], delays in women living in rural areas from seeking care for STI [21], and negative attitudes among HCPs towards STI patients [18], have been shown. Furthermore, previous studies demonstrate that people with STI may seek healthcare from different sources [18] and that HCPs at different levels have very low knowledge and inadequate practice regarding STI, and rarely participate in STI training [20]. Data on the effects of educational interventions regarding STI are limited. The present study was a continuation of the other studies conducted in the same setting [18]-[20]. The aims of this study were to evaluate whether this type of intervention could improve HCPs’ knowledge and reported practice regarding STI in rural Vietnam, and to examine which determinants may possibly impact the intervention’s effectiveness. The ultimate goal was to promote good STI care at grassroots level.

## Methods

### Study setting and participants

This study was a part of a cluster randomised controlled educational intervention conducted in Bavi district, Hanoi, Vietnam in 2010 and 2011. Bavi is a rural district, 60 km west of Hanoi. It covers 410 km^2^ and has a population of approximately 235,000 people within 32 communes. The healthcare system in Bavi district includes one district hospital with 150 beds, three regional polyclinics, 32 commune health centres, village health workers, private facilities and pharmacies/drugstores.

The study participants included medical personnel (medical doctors, assistant medical doctors, nurses and midwives) and pharmacy personnel (pharmacists and drugstore workers) working in public and private facilities in the 32 communes of Bavi district, Hanoi.

### Intervention

**Figure 1:**
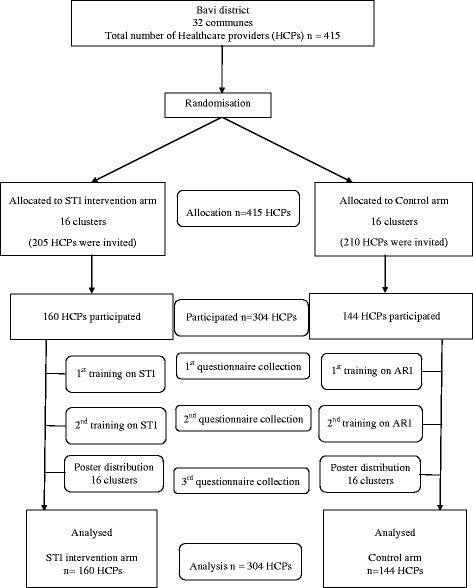
**Flow chart of the study.**

A cluster randomised, controlled trial, with randomisation at commune level, was conducted to examine the effect of a multi-faceted educational intervention on knowledge and reported practice regarding STI and acute respiratory infections (ARI) among HCPs. The 32 communes of Bavi district were randomized into two arms (16 communes per arm), an STI intervention arm and an ARI intervention arm. All HCPs working in the communes were invited to participate. Each arm got active intervention for one topic and functioned as control arm for the other topic (Figure 1). Changes in STI knowledge and reported practice among HCPs from before to after the intervention were assessed through a self-completed questionnaire.

In the STI intervention arm, three activities were performed sequentially from September 2010 to March 2011, including: (i) training session on STI knowledge, (ii) training session on STI case scenario management of four syndromes (see below) based on national guidelines, and (iii) poster distribution to all healthcare facilities in the communes included. In each training session, 25–30 HCPs participated and were divided into four small groups (approximately 5–7 HCPs per group) to discuss different issues related to STI assisted by a trainer (PTL) and facilitator (HDP). Afterwards, each small group presented their group work results, which were then discussed with the entire group. Finally, the trainer gave correct information and a lecture on the topics discussed. For the first training, the issues discussed focussed on STI knowledge, i.e. diseases considered as STI, suspected symptoms, routes of transmission, causes and risk factors, complications, and partner treatment and notification. In the second training, the trainer briefly mentioned the contents of the first training and then divided the HCPs into four small groups to discuss case scenarios of four common STI syndromes including vaginal discharge, urethral discharge, genital ulcers and lower abdominal pain syndromes. The sessions were evaluated using evaluation forms.

Intervention messages used for this study were developed and reviewed by a group of researchers based on the national guidelines. Intervention activities were piloted in two districts outside the study setting. A poster was designed and reviewed by the authors. It comprised short specific messages focusing on STI causes, risk behaviours and complications.

### Questionnaire

The questionnaire included an STI part and an ARI part with the same questions for two arms. However, for the STI intervention arm, the STI part was presented just after questions on HCPs’ characteristics and for the ARI arm, an alternative version of the questionnaire was constructed with the ARI section first, followed by the STI part.

The STI section of the questionnaire was a modified version of questionnaire from a previous study in the same setting [20]. It mainly contained closed and a few open-ended questions on STI knowledge (such as identification of the diseases, suspected symptoms, routes of transmission, causes and risk factors, complications, and partner treatment and notification) and four case scenarios one each for the four syndromes mentioned above.

### Data collection and ethics

The questionnaire was distributed to HCPs just before the first and second training sessions, and one month after poster distribution. The questionnaire was self-completed under the supervision of the research team. No discussion or aid was allowed. All respondents were assured that they could respond anonymously, and that the results would only be used for research purposes. Verbal consent was sought from the respondents before distribution of the questionnaire. The study was approved by the Hanoi Medical University, Vietnam*.*

### Data entry and analyses

ACCESS software was used for data entry, and SPSS version 16 and STATA version 11 for analyses. Proportions, mean, median, minimum and maximum were used for the descriptive analyses. Overall STI knowledge and reported practice was evaluated by scoring. Every correct answer was scored as 1. “Do not know”, incorrect answers or missing responses were given a score of 0. The maximum score one could obtain in the knowledge and reported practice sections of the questionnaire were 56 and 36, respectively. Knowledge and practice improvements were defined by differences of scores after and before the intervention.

Chi-square test was performed to examine the differences between proportions. Cochran test was used for several relative binary samples to verify the proportion trend of correct answers before, during, and after the intervention. Wilcoxon test was used for univariate comparisons of providers’ knowledge and reported practice before and after the intervention. Mann–Whitney test was used to compare knowledge and practice improvements between the STI intervention and control arm. Moreover, multi-level regression analysis was applied to determine which factors influenced the improvements. Intra-cluster correlation (ICC) was used to evaluate the similarity of HCPs within communes regarding STI knowledge and/or practice improvement. Regression coefficients and p-values in T- test, Cochran, Wilcoxon, and Mann–Whitney tests were adjusted for ICC [22].

## Results

Of 415 eligible HCPs, 304 participated in the study, including 160 in the STI intervention arm and 144 in the control arm. The respondents’ mean age was 41.1 years (range 22–69). Most HCPs (89%) had 2–3 years or 6–12 months professional education, and 63% worked at commune health centres. In general, there were no significant differences in socio-demographic characteristics between the HCPs in the STI intervention arm and control arm (p > 0.05). The respondents’ characteristics are shown in Table 1.

**Table 1:** **Socio-demographic characteristics of 304 healthcare providers in the STI intervention arm and control arm**

Category	Intervention arm	Control arm	Total	p-value
	% (n = 160)	% (n = 144)	% (n = 304)	
**Age (years)**				
<35	34	33	34	0.943
35–50	39	41	40	
>50	26	26	26	
**Sex**				
Male	35	33	34	0.664
Female	65	67	66	
**Occupation**				
MD/AMD^a^	59	51	55	0.479
Pharmacist^b^	24	29	27	
Nurse/midwife	16	20	18	
**Working places**				
Commune health centre	68	57	63	0.084
Village level	8	18	12	
Private clinic	5	6	6	
Pharmacy/drugstore	19	19	19	
**Professional education**				
Lower professional training (6–12 months)	12	22	16	0.073
Intermediate professional training (2–3 yrs)	77	67	72	
University/post-graduate	11	11	11	

^a^MD: Medical doctor (15 respondents); AMD: assistant medical doctor (70 respondents).

^b^University pharmacist (2 respondents), intermediate-training pharmacist 3(50 respondents), and low-training pharmacist (27 respondents).

### Intervention and STI knowledge

In general, for almost all questions on STI knowledge, the proportion of correct answers increased significantly in both arms, although HCPs from the STI intervention arm showed a greater increase than their colleagues in the control arm. Gonorrhoea and syphilis were classified as STI by more than 90% of respondents in the two arms both before and after the intervention (Table 2). Less than two thirds of the HCPs in both arms classified trichomonas, genital warts and chlamydia as STI pre-intervention; post-intervention this increased significantly. Compared to before the intervention, the percentage of respondents who classified pubic lice as an STI post-intervention increased significantly in both the STI intervention and the control arm (from 26% to 79% and from 15% to 40%, respectively; p < 0.05). Before the intervention, 70% and 54% of HCPs in the intervention arm classified candidiasis and bacterial vaginosis (BV) as STI, and these were 58% and 37%, in the control arm respectively. After the intervention, a significant improvement to what was observed for candidiasis but not BV in the intervention arm. Conversely, in the control arm, more HCPs classified candidiasis and BV as STI (71% and 59% respectively; p < 0.05) (data not shown).

**Table 2:** **Responses to the questions on STI knowledge among 304 healthcare providers of the two arms before (B), during (D) and after (A) the intervention**

Correct identification		STI intervention arm				Control arm			
		% (n = 160)				% (n = 144)			
		B	D	A	p ^*^	B	D	A	p ^*^
**STI identification**									
	Gonorrhoea	94	98	99	0.119	93	98	99	0.040
	Syphilis	93	96	97	0.312	92	94	99	0.033
	Trichomonas	55	70	89	0.000	51	58	69	0.007
	Genital warts	66	83	93	0.000	63	66	80	0.005
	Chlamydia	40	55	77	0.000	36	49	50	0.019
**Suspected symptoms**									
	Vaginal discharge	70	91	96	0.000	60	72	81	0.001
	Urethral discharge	75	92	98	0.000	72	78	90	0.001
	Genital ulcers, warts	91	95	99	0.027	83	90	92	0.158
	Lower abdominal pain in females	52	65	84	0.000	35	53	62	0.000
**Causes of STI**									
	Bacteria	69	79	75	0.226	60	77	84	0.000
	Viruses	76	74	85	0.106	65	72	88	0.000
	Parasites/protozoa	43	60	77	0.000	43	53	56	0.088
**Risk factors for STI**									
	Unsafe sex	91	93	100	0.005	87	91	97	0.010
	Unsafe blood transfusion	40	61	75	0.000	29	38	51	0.000
**Routes of transmission**									
	Sexual intercourse	97	93	99	0.106	97	100	100	0.121
	Blood transfusion/ needle sharing	51	71	81	0.000	44	58	60	0.016
	Mother-to-child	64	83	93	0.000	56	56	76	0.000
**Necessity of partner examination/treatment**									
	Gonorrhoea	96	97	99	0.310	95	97	100	0.047
	Syphilis	96	96	99	0.284	93	96	99	0.028
	Trichomonas	62	77	86	0.000	63	63	65	0.887
	Genital warts	82	86	96	0.017	77	69	76	0.231
	Chlamydia	50	64	78	0.000	47	46	54	0.292
**Complications of STI if untreated**									
	Infertility	96	95	99	0.333	88	95	94	0.052
	Ectopic pregnancy/cervical cancer	81	92	93	0.022	65	85	87	0.000
	Adverse pregnancy outcome	85	92	98	0.004	71	90	95	0.000
	Pelvic inflammation	52	65	79	0.000	36	50	49	0.017
**Ways of STI prevention**									
	Save sex including condom use	98	99	98	0.822	94	98	100	0.009
	Being faithful**	93	96	97	0.186	81	90	97	0.000

^*^Cochran test for verifying the proportion trend of correct answers before, during and after the intervention, with p-value adjusted for ICC.

^**^Maintaining sexual loyalty to one's spouse or lover.

Concerning knowledge about partner notification for patients who suffered from trichomonas, genital warts or chlamydia, Table 2 shows significant improvements among HCPs in the intervention arm but not the control arm. Furthermore, pre-intervention, 71% and 81% of the HCPs in the STI intervention arm answered the necessity of partner notification and treatment for BV and candidiasis, and these were significantly reduced post-intervention (57% and 69%). In contrast, no significant differences knowledge on partner treatment for these two infections were observed in the control arm (data not shown).

Regarding risk factors, there was a decreased number of HCPs in the intervention arm who answered that a risk factor for STI was bad hygiene (62% before, 45% after; p < 0.05), while the number of providers who answered that having sex during menstruation posed a risk was almost unchanged (55% before, 58% after; p > 0.05). In contrast, more HCPs in the control arm responded that bad hygiene (66% before, 69% after; p > 0.05), or having sex during menstruation (37% before, 56% after; p < 0.05) were risk factors (data not shown).

Table 3 shows the improvement of STI knowledge in the two arms. Overall, after the intervention, the mean knowledge scores of most of the HCPs in both arms increased significantly (p < 0.05) and HCPs in the STI intervention arm presented higher mean knowledge scores than their colleagues in the control arm. The total mean knowledge score of the respondents in the STI intervention arm pre-intervention was 37.2 (range 12–53; median 38), and 48.4 post-intervention (range 19–56; median 50); for the control group it was 32.7 (range 0–53; median 33) and 41.7 (range 13–56; median 44), respectively. The improvement observed in both arms was statistically significant (p < 0.05). The proportion of HCPs that had positive knowledge improvement was 91% in the STI intervention arm and 80% in the control arm (p = 0.004). The ICCs of knowledge improvement among HCPs within communes were 0.21 in the STI intervention arm and 0.16 in the control arm.

**Table 3:** **Univariate comparison of knowledge improvement between the STI intervention arm and control arm**

		STI intervention arm				Control arm				
		Mean score (n = 160)				Mean score (n = 144)				
Category		Before	After	p ^*^	Difference	Before	After	p ^*^	Difference	p ^**^
**Sex**										
	Male	37.4	48.7	0.000	11.3	33.0	41.1	0.000	8.0	0.261
	Female	37.2	48.2	0.000	11.1	32.51	42.0	0.000	9.5	0.212
**Age** (years)										
	<35	37.4	48.5	0.000	11.0	34.0	42.1	0.000	8.1	0.223
	35-50	35.6	48.5	0.000	12.9	34.0	43.3	0.000	9.4	0.107
	>50	39.5	48.2	0.000	8.7	29.1	38.7	0.000	9.6	0.694
**Profesional education**										
	Lower training (6–12 months)	32.5	43.8	0.002	11.3	23.7	36.0	0.000	12.3	0.897
	Intermediate training (2–3 yrs)	37.3	48.8	0.000	11.5	34.9	43.8	0.000	8.8	0.029
	University/post-graduate	41.8	50.3	0.001	8.6	36.4	40.3	0.044	3.9	0.261
**Occupation**										
	MD/AMD^a^	38.8	48.5	0.000	9.7	37.4	43.6	0.000	6.2	0.034
	Pharmacist	37.4	48.0	0.000	10.6	28.0	37.7	0.000	9.7	0.638
	Nurse/midwife	33.3	48.3	0.000	15.0	30.7	42.6	0.000	11.9	0.317
**Working places**										
	Commune health centre	38.9	48.7	0.000	9.8	37.0	44.1	0.000	7.2	0.082
	Village level	29.6	46.2	0.001	16.5	21.7	38.0	0.000	16.3	0.987
	Private clinic	41.4	51.1	0.012	9.8	21.8	27.6	0.091	5.9	0.154
	Pharmacy/drugstore	33.5	47.9	0.000	14.4	32.0	41.7	0.000	9.7	0.154
**Total scores**										
	Mean	37.2	48.4	0.000	11.2	32.7	41.7	0.000	9.0	0.207
	Median	38	50	0.000	11	33	44	0.000	8	0.142
	Minimum	12	19		−14	0	13		−10	
	Maximum	53	56		40	53	56		46	

^*^ Wilcoxon test (T-test for mean of total scores) to compare related samples (before and after intervention) of each arm, with p-value adjusted for ICC.

^**^Mann–Whitney test (T-test for mean of total scores) to compare knowledge scores differences (after - before) between the intervention and the control arms, with p-value adjusted for ICC.

^a^MD: Medical doctor AMD: assistant medical doctor.

When comparing by HCPs’ characteristics between the STI intervention and control arms, a significantly higher improvement in knowledge scores was observed in the STI intervention arm among HCPs who had intermediate professional training, and medical doctors or assistant medical doctors (p < 0.05).

### Intervention and STI reported practice

In general, after the intervention, the reported practice scores of most of HCPs in both arms increased significantly (p < 0.05) and HCPs in the STI intervention arm presented higher scores than their colleagues in the control arm. Average reported practice score of the respondents in the STI intervention arm before the intervention was 17.6 (range 1–28, median 18), and after the intervention 21.8 (range 0–34; median 23); for the control arm it was 14.8 (range 1–26; median 16) and 18.5 (range 3–33; median 19), respectively. The improvement observed in both arms was statistically significant (p < 0.05). The difference in average scores before and after the intervention (after - before) in the STI intervention arm and control arm was 4.3 and 3.6, respectively, but this difference was not statistically significant (p > 0.05). The proportion of HCPs that had positive practice improvement was 72% in the STI intervention arm and 65% in the control arm (p = 0.349). The ICCs of practice improvement among HCPs within communes were for 0.20 in the STI intervention arm and 0.07 in the control arm.

When comparing the differences in the mean scores after and before the intervention between the two arms by each category of participants (Table 4), the results showed significantly higher improvement in the STI intervention arm, only among HCPs with intermediate professional training.

**Table 4:** **Univariate comparison of practice improvement between the STI intervention arm and control arm**

		STI intervention arm				Control arm				
		Mean score (n = 160)				Mean score (n = 144)				
Category		Before	After	p ^*^	Difference	Before	After	p ^*^	Difference	p ^**^
**Sex**										
	Male	18.9	21.9	0.003	2.9	14.6	18.4	0.000	3.8	0. 857
	Female	16.8	21.8	0.001	5.0	14.9	18.5	0.000	3.6	0.228
**Age** (years)										
	<35	16.9	22.0	0.001	5.2	14.8	18.5	0.002	3.6	0.442
	35-50	16.9	22.4	0.000	5.5	16.3	19.8	0.001	3.4	0.052
	>50	19.4	20.7	0.149	1.3	12.5	16.5	0.004	4.0	0.291
**Education**										
	Lower training (6–12 months)	16.0	16.2	0.892	0.2	13.5	16.6	0.002	3.2	0.358
	Intermediate training (2–3 yrs)	17.5	22.6	0.000	5.2	15.3	19.1	0.000	3.8	0.047
	University/post-graduate	19.6	22.2	0.159	2.6	14.6	18.3	0.008	3.8	0.767
**Occupation**										
	MD/AMD^a^	18.2	22.3	0.000	4.1	15.7	19.6	0.000	3.9	0.494
	Nurse/midwife	17.8	21.1	0.079	3.3	13.8	17.0	0.018	3.3	0.936
	Pharmacist	15.7	21.1	0.014	5.3	14.4	18.1	0.000	3.7	0.279
**Working places**										
	Commune health centre	18.2	22.2	0.001	4.0	16.0	19.6	0.001	3.6	0.386
	Village level	18.1	18.7	0.503	0.6	13.6	17.6	0.002	3.9	0.184
	Private clinic	18.0	22.0	0.024	4.0	11.3	13.0	0.445	1.8	0.173
	Pharmacy/drugstore	14.8	21.7	0.002	6.8	13.6	18.2	0.001	4.6	0.173
**Total scores**										
	Mean	17.6	21.8	0.000	4.3	14.8	18.5	0.000	3.6	0.581
	Median	18	23	0.000	4	16	19	0.000	3	0.388
	Minimum	1	0		−21	1	3		−12	
	Maximum	28	34		29	26	33		22	

^*^Wilcoxon test (T-test for mean of total scores) to compare related samples (before and after intervention) of each arm, with p-value adjusted for ICC.

^**^Mann–Whitney test (T-test for mean of total scores) to compare practice scores differences (after - before) between the STI intervention and the control arm, with p-value adjusted for ICC.

^a^MD: Medical doctor; AMD: assistant medical doctor.

### Factors influencing the interventions’ effects

Table 5 presents the improvement of HCPs’ STI knowledge and reported practice using multi-level multivariate regression analysis. The improvement of both STI knowledge and reported practice was not significant in reference categories (regression constants were 0.78 and −0.99; p > 0.05). However, being aged between 35 and 50 years, having intermediate professional training, being a pharmacist, working at village level, or being part of the STI intervention arm, were significant determinants which added 2.81, 4.43, 5.53, 7.91, or 2.97 points, respectively (p < 0.05) to the improvement of HCPs’ STI knowledge.

**Table 5:** **Knowledge and practice improvement in multi-level multivariate regression analysis**

	Knowledge improvement		Practice improvement	
	Regression coefficient ^*^	p-value	Regression coefficient ^*^	p-value
**Participation in the STI intervention**				
No	0	Reference	0	Reference
Yes	2.97	0.008	0.33	0.688
**Age** (years)				
<35	0.35	0.795	2.00	0.075
35-50	2.81	0.028	2.15	0.042
>50	0	Reference	0	Reference
**Professional education**				
Lower training (6–12 months)	0	Reference	0	Reference
Intermediate training (2–3 yrs)	4.43	0.006	3.33	0.014
University/Postgraduate	1.72	0.424	2.26	0.207
**Occupation**				
MD/AMD^a^	0	Reference	0	Reference
Nurse/midwife	1.60	0.263	−0.36	0.756
Pharmacist	5.53	0.001	−0.73	0.580
**Working places**				
Commune health centrer	0	Reference	0	Reference
Village level	7.91	0.000	1.65	0.299
Private clinic	2.22	0.347	0.96	0.620
Pharmacy/drugstore	0.73	0.671	3.22	0.024
Constant	0.78	0.718	−0.99	0.573

^*^Regression coefficients were adjusted for intra-cluster correlations.

^a^MD: Medical doctor; AMD: assistant medical doctor.

Concerning HCPs’ reported practice, being aged between 35 and 50 years, having intermediate professional training, or working at a pharmacy/drugstore, were significant determinants which added 2.15, 3.33, or 3.22 points (p < 0.05).

## Discussion

In this study we found that HCPs who received an educational intervention on STI were more likely to have improved STI knowledge and reported practice than those in the control arm, though a significant different in reported practice was not found in multivariate analysis. The study also identified several determinants influencing the interventions’ effectiveness.

Our results showed that the number of correct answers increased after each educational activity, both in the intervention as well as the control arm, and that the majority of the improvements were statistically significant. This improvement could be due to the impact of the intervention because HCPs in the intervention arm showed a greater increase than their colleagues in the control arm. Moreover, for issues where we considered initial knowledge low, such as STI identification and partner treatment for pubic lice, genital herpes/warts, and chlamydia, improved much more among HCPs in STI intervention arm as compared to that of the control arm. On the other hand, misconceptions on the definition of STI and partner treatment for BV and candidiasis, and risk factors of STI, decreased among HCPs in the STI intervention arm, and increased in the control arm.

Surprisingly, more than 60% of the HCPs in both arms considered partner treatment necessary for BV and candidiasis, and also, more than half believed that bad hygiene or sex during menses were risk factors for STI. After the intervention, the misconceptions were reduced but remained common. Previous studies conducted in the same setting, also show these misconceptions among HCPs [20], as well as among people in the community [18],[19]. After several years, these misconceptions have remained mostly unchanged, though HCPs’ knowledge in the present study was better as compared to the earlier study [20]. This implies the need to provide educational activities that focus not only on knowledge, but also misconceptions.

The ICCs in the STI intervention arm can be considered high, which indicates high similarities of the HCPs within communes regarding the improvements of both knowledge and practice. This may be due to discussions on STI among trained HCPs within communes after each educational activity. Lower values of ICCs in the control arm might indicate less emphasising STI discussions among HCPs within communes, especially issues related to written case scenarios (reported practice).

We considered that the intervention activities might provide impacts on knowledge and practice improvement. The impacts could be produced by: i) training/education activities, ii) repeated filling of the questionnaires, and iii) possible discussions among HCPs on the related topic. Multi-level multivariate regression analysis showed significant impact of several factors, including being part of the STI intervention, age, professional education, occupation or working place, on knowledge improvement. Among these factors, being part of the STI intervention contributed 3 points to knowledge improvement.

Randomised controlled studies have shown positive effects of educational interventions on improving HCPs’ practice [23] or self-efficacy to perform a certain task [24],[25] especially when taking multi-component interventions into account, including a simulated client method [26],[27]. Our study did not show significant direct impacts of the STI intervention on HCPs’ reported practice, as shown elsewhere [28]. However, our study did show that, indirect impacts of the intervention on practice improvement were significant in specific groups based on, age, professional education, and working place.

Notably, in the multivariate analysis models, our results showed significant knowledge improvement among pharmacists and reported practice, among those who worked at pharmacies/drugstores. Studies have also shown that educational interventions improve pharmacy workers’ practice [26],[29], syndromic management of STI at pharmacies [30], and cost-effectiveness of syndromic management of STI though pharmacist training [31]. Studies in Vietnam and elsewhere show that STI patients usually go to pharmacies/drugstores for treatment or advice [18],[32]-[34] because of the lower costs, higher accessibility [18],[34] and less stigmatisation [18]. Moreover, the majority of pharmacies treat STI patients [35] therefore training of pharmacy workers can improve antimicrobial prescribing practices for STI syndrome management in Vietnam [36]. Thus improving knowledge and practice for pharmacists/drug sellers may play an important role in STI control and prevention strategies, and it cannot be ignored.

### Methodological considerations

A consensus about the types of situations in which the contamination of educational interventions is more or less likely has been shown [37], and cluster randomisation may reduce contamination [38]. However, in our study, prevention of contamination could not be entirely assured due to the fact that HCPs in different communes could meet each other on various occasions where they might have exchanged information, including issues related to STI. Therefore, the improvement in the control group could partially have been a consequence of contamination. Subsequently, the impact of the STI intervention might actually be higher than presented in our results. Further, reported practice of HCPs might not reflect their actual practice due to lack of information provided in the scenarios, which might cause biases in the evaluation of practice improvement.

A point which might be raised here is that, asking questions repetitively can automatically result in improvements. Possibly, completing the questionnaire three times made the HCPs familiar with the questions asked, which made their responses easier the subsequent times. It could also be assumed that filling in the questionnaire several times is an intervention in itself. Moreover, possible discussions about STI among HCPs in both arms after each training session could be a potential influencing factor.

The randomised design can be considered the best protection against confounding and selection bias [39]. By choosing a randomised controlled design, the intention of producing comparable groups of HCPs who differ only in terms of their exposure to intervention during the study was assured, and several potential confounders or biases, such as geographical location, HCPs’ professional education and occupation, could be removed. However, due to the fact that the study was carried out in a rural district of Vietnam, the results of this study may be considered to be representative for only HCPs working at similar grassroots healthcare settings.

We found that the initial proportions of correct answers and mean knowledge scores in the intervention arm were higher as compared to those in the control arm. This could be due to the difference in the order of questions related to STI and ARI within the questionnaires used for each arm. Moreover, HCPs may have placed more attention on the topic they were trained in. Consequently, they could have correctly answered more questions related to the main topic of the arm in which they participated. However, the above mentioned effects did not influence our results since the improvements were calculated as the differences between pre- and post- intervention scores.

We also analysed the impact of each educational activity on STI knowledge and reported practice and found that, the differences of STI knowledge before and after the first training, as well as the second training, were not significant (results not shown). However, knowledge improvement in the whole intervention process was significant. While, practice training was given only once in our study, which might possibly have influenced the impact of the STI intervention on practice improvement. Hence, multi-faceted interventions have been shown to be effective in particular environments [26],[36]. We used an interactive training method for each training session, which is not commonly applied in our educational system, as well as training in the field. This method is considered to motivate active learning from participants and provide better training effects, and can result in moderately large changes in practice, while didactic lectures alone are unlikely to affect change [40].

## Conclusion

This study shows the effectiveness of an educational intervention on STI knowledge of HCPs at grassroots level, especially for pharmacists, HCPs who worked in villages, and who initially had low STI knowledge. Our study does not show the significant effect of intervention on practice overall, however the significant effect was observed among pharmacy/drugstore workers. Interactive training and multi-faceted educational interventions may give better and more sustainable improvements, and the education curriculum should not only focus on providing correct knowledge but also address misconceptions.

## Authors’ original submitted files for images

Below are the links to the authors’ original submitted files for images. Authors’ original file for figure 1
