# Supplementary figures and images for: Improved knowledge and reported practice regarding sexually transmitted infections among healthcare providers in rural Vietnam: a cluster randomised controlled educational intervention
Source: BMC Infect Dis. 2014 Dec 4;14:646. doi: 10.1186/s12879-014-0646-5 (PMC4263008; doi:10.1186/s12879-014-0646-5)

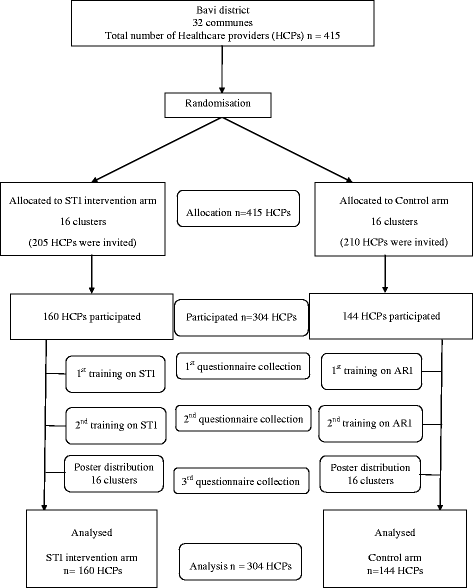

Supplement: Supplementary file 1 — Authors’ original file for figure 1 [file 12879_2014_646_MOESM1_ESM.gif]
